# Artificial Intelligence-Enhanced Liquid Biopsy and Radiomics in Early-Stage Lung Cancer Detection: A Precision Oncology Paradigm

**DOI:** 10.3390/cancers17193165

**Published:** 2025-09-29

**Authors:** Swathi Priya Cherukuri, Anmolpreet Kaur, Bipasha Goyal, Hanisha Reddy Kukunoor, Areesh Fatima Sahito, Pratyush Sachdeva, Gayathri Yerrapragada, Poonguzhali Elangovan, Mohammed Naveed Shariff, Thangeswaran Natarajan, Jayarajasekaran Janarthanan, Samuel Richard, Shakthidevi Pallikaranai Venkatesaprasath, Shiva Sankari Karuppiah, Vivek N. Iyer, Scott A. Helgeson, Shivaram P. Arunachalam

**Affiliations:** 1 Digital Engineering & Artificial Intelligence Laboratory (DEAL), Mayo Clinic, Jacksonville, FL 32224, USA; swathicherukuri13@gmail.com (S.P.C.); anmolpreetkgrewal@gmail.com (A.K.); bipashagoyal@gmail.com (B.G.); drhanisha3391@gmail.com (H.R.K.); mehrishmurtazasahito@gmail.com (A.F.S.); gayathriy9322@gmail.com (G.Y.); mohammednaveedshariff.r@gmail.com (M.N.S.); thangeswarann@gmail.com (T.N.); the.samuel.richard@gmail.com (S.R.);; 2 Department of Internal Medicine, McLaren, Bay City, MI 48706, USA; pratyushsachdeva1@gmail.com; 3 Division of Pulmonary & Critical Care Medicine, Department of Medicine, Mayo Clinic, Rochester, MN 55905, USA; 4 Division of Pulmonology, Department of Medicine, Mayo Clinic, Jacksonville, FL 32224, USA; 5 Department of Critical Care Medicine, Mayo Clinic, Jacksonville, FL 32224, USA

**Keywords:** lung cancer, liquid biopsy, artificial intelligence, radiomics, early detection, precision oncology

## Abstract

**Simple Summary:**

Lung cancer is one of the deadliest cancers because it is often found too late. Current tests like CT scans or tissue biopsies can miss early signs and may be risky or uncomfortable for patients. A newer, safer method called liquid biopsy uses a blood sample to look for cancer-related materials. However, it’s not always accurate in early stages. In this study, we explore how artificial intelligence (AI) and imaging tools like radiomics can help improve liquid biopsy accuracy. By combining blood test data, medical images, and computer models, doctors may be able to detect lung cancer earlier and choose the right treatment faster. This new approach could reduce deaths and make cancer care more personal and less invasive.

**Abstract:**

Background: Lung cancer remains the leading cause of cancer-related mortality globally, largely due to delayed diagnosis in its early stages. While conventional diagnostic tools like low-dose CT and tissue biopsy are routinely used, they suffer from limitations including invasiveness, radiation exposure, cost, and limited sensitivity for early-stage detection. Liquid biopsy, a minimally invasive alternative that captures circulating tumor-derived biomarkers such as ctDNA, cfRNA, and exosomes from body fluids, offers promising diagnostic potential—yet its sensitivity in early disease remains suboptimal. Recent advances in Artificial Intelligence (AI) and radiomics are poised to bridge this gap. Objective: This review aims to explore how AI, in combination with radiomics, enhances the diagnostic capabilities of liquid biopsy for early detection of lung cancer and facilitates personalized monitoring strategies. Content Overview: We begin by outlining the molecular heterogeneity of lung cancer, emphasizing the need for earlier, more accurate detection strategies. The discussion then transitions into liquid biopsy and its key analytes, followed by an in-depth overview of AI techniques—including machine learning (e.g., SVMs, Random Forest) and deep learning models (e.g., CNNs, RNNs, GANs)—that enable robust pattern recognition across multi-omics datasets. The role of radiomics, which quantitatively extracts spatial and morphological features from imaging modalities such as CT and PET, is explored in conjunction with AI to provide an integrative, multimodal approach. This convergence supports the broader vision of precision medicine by integrating omics data, imaging, and electronic health records. Discussion: The synergy between AI, liquid biopsy, and radiomics signifies a shift from traditional diagnostics toward dynamic, patient-specific decision-making. Radiomics contributes spatial information, while AI improves pattern detection and predictive modeling. Despite these advancements, challenges remain—including data standardization, limited annotated datasets, the interpretability of deep learning models, and ethical considerations. A push toward rigorous validation and multimodal AI frameworks is necessary to facilitate clinical adoption. Conclusion: The integration of AI with liquid biopsy and radiomics holds transformative potential for early lung cancer detection. This non-invasive, scalable, and individualized diagnostic paradigm could significantly reduce lung cancer mortality through timely and targeted interventions. As technology and regulatory pathways mature, collaborative research is crucial to standardize methodologies and translate this innovation into routine clinical practice.

## 1. Introduction

Lung cancer remains the leading cause of cancer-related mortality worldwide, accounting for approximately 1.8 million deaths annually [1]. It is broadly classified into non-small cell lung cancer (NSCLC, ~85%) and small cell lung cancer (SCLC). NSCLC is driven by oncogenic alterations, including mutations in EGFR (10–30%), KRAS (15–30%), and ALK rearrangements (3%), among others [2]. The high mortality rate is largely attributed to late-stage diagnosis, occurring in nearly 70% of patients [3]. In contrast, early-stage lung cancer carries a 5-year survival rate exceeding 60–70% [4], underscoring the urgent need for effective early detection strategies.

Low-dose computed tomography (LDCT) is the primary screening tool for high-risk individuals (aged 55–80 with significant smoking histories) [5]. Landmark trials, including the National Lung Screening Trial (NLST) and the NELSON study, demonstrated reduced mortality with LDCT [6]. However, LDCT has notable drawbacks, including high false-positive rates, radiation exposure, limited accessibility in low-resource settings, and reduced utility in non-smokers [7]. Moreover, the frequent detection of indeterminate nodules often triggers unnecessary procedures and psychological stress, adding to healthcare costs [8].

Tissue biopsy remains the gold standard for diagnosis and histological subtyping, but its invasiveness, cost, sampling error, and procedural risks limit its use in routine or longitudinal monitoring [9]. Additionally, Tumor evolution and acquired resistance also reduce the reliability of a single tissue sample, highlighting the need for more dynamic approaches.

Liquid biopsy, a non-invasive alternative, analyzes tumor-derived components such as circulating tumor DNA (ctDNA), cell-free DNA (cfDNA), circulating tumor cells (CTCs), exosomes, and microRNAs in body fluids like blood or urine [10]. It offers real-time monitoring of tumor dynamics and better reflects tumor heterogeneity while minimizing patient risk [11]. Although liquid biopsy is promising for early detection and molecular profiling, sensitivity and specificity remain suboptimal in early-stage disease [12,13].

Recently, Artificial intelligence (AI) has strengthened liquid biopsy by enabling the integration of genomic, epigenomic, and proteomic data for improved detection, prognostication, and treatment guidance [14]. Within this broader AI field, bio-inspired optimization methods such as the Whale Optimization Algorithm (WOA) have shown utility mainly in imaging workflows for feature selection and classifier tuning (e.g., WOA–SVM; multispiral WOA–APSO) [15,16]. These results are encouraging but limited by small, retrospective datasets and a lack of prospective validation, so their role in liquid biopsy pipelines remains uncertain.

Accordingly, this review focuses on AI-enabled liquid biopsy for early lung cancer detection, outlining recent progress, persistent challenges, and future directions.

## 2. Methods

### 2.1. Review Design

We conducted a focused narrative review with structured search and screening to synthesize evidence on AI-enabled liquid biopsy for lung cancer and on integration with imaging/radiomics and LDCT. Where feasible, we mapped study design, cohorts, assays, and validation strategies to highlight translational readiness and gaps (e.g., generalizability, head-to-head comparisons with standard modalities).

### 2.2. Information Sources and Search Strategy

Electronic databases (PubMed/MEDLINE, Embase, Scopus, Web of Science) and IEEE Xplore (for AI/ML methods and radiomics) were searched from inception through March 2025. Additional sources included the Cochrane Library (for screening/diagnostics), Google Scholar (for citation chasing), and selected preprint servers (arXiv, medRxiv), with preference for peer-reviewed versions when available.

Search terms combined keywords related to lung cancer, liquid biopsy, and artificial intelligence. Examples included:

“lung cancer” OR “non-small cell lung cancer” OR “NSCLC” OR “small cell lung cancer” OR “SCLC”

“liquid biopsy” OR “circulating tumor DNA” OR “ctDNA” OR “cell-free DNA” OR “cfDNA” OR “circulating tumor cells” OR “CTCs” OR “exosomes” OR “extracellular vesicles”

“artificial intelligence” OR “machine learning” OR “deep learning” OR “neural networks” OR “radiomics” OR “bioinformatics”

These terms were applied in varying combinations across databases. Reference lists of eligible articles and key reviews were also screened. Only English-language publications were included.

### 2.3. Inclusion and Exclusion Criteria

Inclusion:

Original research or systematic reviews on AI applied to liquid biopsy in lung cancer.

Studies describing diagnostic, prognostic, predictive, or monitoring applications.

Articles reporting methodological details of AI models, biomarkers, or datasets.

Exclusion:

Case reports, conference abstracts without peer-reviewed full texts, editorials, and letters.

Studies not involving lung cancer or not applying AI to liquid biopsy-related biomarkers.

Articles lacking sufficient methodological or outcome data.

Study Selection and Data Extraction

Two reviewers independently screened all retrieved records at the title/abstract level, followed by full-text review for eligibility. Discrepancies were resolved by consensus. For each included study, data were extracted on study design, patient population, type of liquid biopsy biomarker (ctDNA, cfDNA, CTCs, exosomes, RNA, proteins), AI methodology, key outcomes (e.g., sensitivity, specificity, AUC), and reported limitations. Given the narrative scope, we did not apply formal risk-of-bias tools such as QUADAS-2 or PROBAST. Instead, we qualitatively appraised methodological rigor and reported limitations of each study, focusing on aspects such as sample size, validation approach, and generalizability.

## 3. Lung Cancer Overview

### 3.1. Epidemiology

Lung cancer is the second most common cancer in the U.S. and the leading cause of cancer-related deaths globally [17]. High mortality reflects frequent late-stage presentation, as symptoms such as cough, hemoptysis, and dyspnea are nonspecific [18]. Smoking is the most significant risk factor, although additional contributors include secondhand smoke, genetic predisposition, environmental exposures like radon and asbestos, and underlying pulmonary conditions such as COPD and interstitial lung disease [19]. Importantly, 10–25% of lung cancers occur in never-smokers, ranking as the fifth leading cause of cancer deaths in this population [20].

### 3.2. Types of Lung Cancer

Histologically, lung cancer is categorized into two primary subtypes: small cell lung cancer (SCLC) and non-small cell lung cancer (NSCLC) [21].

Small Cell Lung Cancer (SCLC): SCLC accounts for 15–20% of cases, is neuroendocrine in origin, and is defined by rapid proliferation and early metastasis [22]. It predominantly affects current or former smokers, typically with a latency of ~30 years [23]. While symptoms are non-specific, Imaging often shows central thoracic masses with bulky lymphadenopathy. Genetically, >90% of cases harbor TP53 and RB1 loss, alongside alterations in MYC, PTEN, NOTCH, and CREBBP, which regulate chromatin structure and DNA repair [22,23]. SCLC demonstrates lineage plasticity, complicating therapy, while transcription factors such as Nuclear Factor I B (NFIB) enhance chromatin accessibility and promote metastatic behavior [24]. SCLC subtypes are defined by dominant transcription factors (Table 1).

Median survival remains ~2 years for limited-stage and ~1 year for extensive-stage disease despite chemotherapy, emphasizing the need for early detection [26].

Non-Small Cell Lung Cancer (NSCLC): NSCLC represents 80–85% of cases, including adenocarcinoma, squamous cell carcinoma, large-cell carcinoma, and bronchial carcinoids, with adenocarcinoma being the most prevalent [21]. It is driven by oncogenic alterations activating RAS–RAF–MEK–ERK and PI3K–AKT–mTOR pathways, promoting uncontrolled cell proliferation, survival, and immune evasion [27]. Common mutations and molecular targets in NSCLC are discussed in Table 2.

### 3.3. Current Screening and Diagnostic Approaches

Annual low-dose computed tomography (LDCT) screening is recommended only for high-risk individuals. The 2013 U.S. Preventive Services Task Force (USPSTF) guidelines defined eligibility as 55–80 years with ≥30 pack-years, including former smokers. In 2021, this was broadened to 50 years with ≥20 pack-years, thereby expanding access and minimizing missed cases [29]. However, these criteria still overlook important factors such as gender, socioeconomic status, geography, and ethnicity [30]. Outside the U.S., adoption of LDCT screening remains limited, with several European and Asian countries conducting pilot programs but no universal population-wide implementation.

Even among eligible individuals, uptake is low (~16.5% in 2022) due to fear of false positives, invasive follow-up, and stigma [31]. False positives are especially problematic in regions endemic for granulomatous infections like tuberculosis, which can mimic malignancy radiographically [32]. Furthermore, current screening detects only about 50% of lung cancers [33]. Traditional tools such as chest X-ray and sputum cytology are insensitive. While hnRNP A2/B1 (heterogeneous nuclear ribonucleoprotein A2/B1, a nuclear protein involved in RNA splicing, whose overexpression has been linked to early carcinogenesis in lung cancer) immunostaining offers better detection, it is not widely adopted [34,35].

Definitive diagnosis requires tissue sampling via bronchoscopy, CT-guided needle biopsy, or surgical biopsy. While white light bronchoscopy is standard, its sensitivity is low for small or premalignant lesions. Techniques such as Endobronchial ultrasound (EBUS) and fluorescence bronchoscopy offer enhanced sensitivity for central tumors and lymph node evaluation, but remain limited for dysplasia [36].

Simultaneously, Molecular analysis is critical in advanced NSCLC for actionable mutations (EGFR, ALK, ROS1, BRAF), yet tissue yield and procedural risk often limit comprehensive genomic analysis [12].

To overcome these limitations, Liquid biopsy is emerging as a non-invasive solution, detecting analytes such as ctDNA, CTCs, exosomes, and miRNAs. Even stage IA cancers show elevated cfDNA compared to healthy controls [37,38]. CTCs provide prognostic and therapeutic insights and can now be detected with high precision using markers like EpCAM. However, ctDNA falls sharply after surgery, limiting post-op surveillance [13].

Aside from cfDNA and CTCs, Other promising biomarkers include hypermethylated p16 and lung-specific miRNAs in sputum, which can distinguish adenocarcinoma from squamous carcinoma. Though still experimental, these markers offer future potential for early, non-invasive diagnosis [39,40].

## 4. Liquid Biopsy in Lung Cancer

The term “liquid biopsy” was introduced in 2010 when circulating tumor cells (CTCs) were first identified in cancer patients [41]. It refers to the sampling of non-solid biological tissue, especially body fluids like blood, for cancer detection. By definition, a liquid biopsy is a minimally invasive method of collecting biological material to identify tumor cells or associated biomarkers [42]. Unlike traditional biopsies that often require surgical intervention, liquid biopsy typically involves a simple blood or urine sample. This significantly reduces the risk of complications, enables convenient repeat sampling for longitudinal monitoring, and allows multi-site analysis to better capture tumor heterogeneity—all at a lower cost [43].

**Figure 1:**
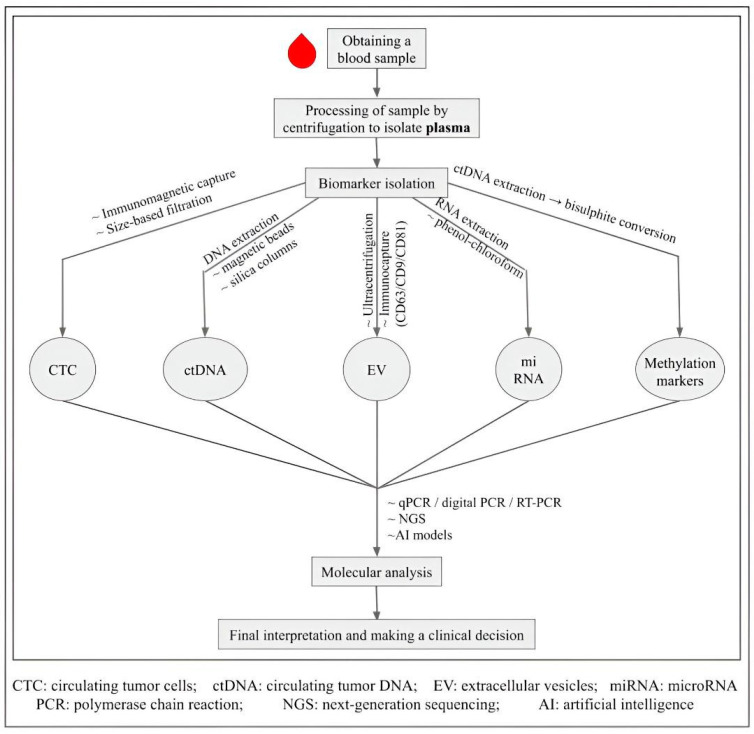
Workflow of liquid biopsy in lung cancer.

The process begins with a blood sample, which is centrifuged to isolate plasma. Tumor-derived biomarkers—including CTCs, circulating tumor DNA (ctDNA), extracellular vesicles (EVs), microRNAs, and DNA methylation markers—are then isolated using specialized techniques. These analytes undergo molecular analysis (qPCR, digital PCR, next-generation sequencing) and increasingly AI-based models to support clinical decision-making, as illustrated in Figure 1.

### 4.1. Types of Biomarkers in Liquid Biopsy

Liquid biopsy detects biomarkers shed by tumors through apoptosis, necrosis, or secretion. Key biomarkers include:

1. Circulating Tumor Cells (CTCs): These are tumor-derived cells that enter the bloodstream from the primary or metastatic site of the tumor. Although they are present in low concentrations, CTCs offer molecular information (DNA, RNA, protein) and correlate with metastasis. Higher expression of genes such as PIK3CA, AKT2, TWIST, and ALDH1 in CTCs is seen in patients with metastatic lung cancer compared to patients with non-metastatic disease [44].
2. Circulating tumor DNA (ctDNA): Cell-free DNA (cfDNA) is defined as non-encapsulated, fragmented DNA found in the blood of an individual. While present in healthy individuals, levels are significantly higher in cancer patients due to increased apoptosis, necrosis, and active secretion from proliferating tumor cells [45]. Plasma is preferred over serum for cfDNA analysis, as serum collection induces leukocyte lysis during clotting, releasing high amounts of genomic DNA that contaminate cfDNA and compromise assay sensitivity [46]. The concentration of circulating tumor DNA (ctDNA) can be extremely low and difficult to detect, but its detection can prove to be beneficial as the molecular alterations present in ctDNA closely resemble the tumor tissue [47,48]. Many techniques are being employed to detect ctDNA with high sensitivity and specificity, including droplet digital PCR, next-generation sequencing, Sanger sequencing, etc. CtDNA is an important biomarker as it can help detect various mutations responsible for the tumor and guide the treatment accordingly [49].
3. Extracellular vesicles (EV): Extracellular vesicles are nano-sized, membrane-bound particles released into the extracellular space. The three main subtypes of extracellular vesicles are: microvesicles, exosomes, and apoptotic bodies, which can be distinguished based on multiple factors like their biogenesis, release pathways, size, content, and function [50]. Cancer cells secrete more EVs than normal cells, which promote tumor progression and metastasis through angiogenesis and immune evasion, making it a potential biomarker [51,52]. Exosomal miRNA (exo-miRNA) is another key prognostic biomarker that is being explored and can be easily detected in the bloodstream, as it is protected from degradation by a lipid bilayer [11,53].
4. MicroRNAs (miRNAs): Small, non-coding RNAs that regulate gene expression and are found circulating in the body fluids of cancer patients. The microRNAs let-7i-3p and miR-154-5p serve as diagnostic and prognostic biomarkers in lung cancer. They belong to tumor suppressor miRNA families and are downregulated in lung cancer. Low levels of these microRNAs detected via liquid biopsy indicate poor prognosis [54].
5. DNA Methylation Markers: Aberrant methylation of CpG islands is common in cancer. Profiling tools such as bisulfite conversion and high-density methylation arrays (e.g., HumanMethylationEPIC BeadChip) are powerful screening and prognostic platforms [55].
6. Tumor-educated platelets (TEPs): Platelets reprogrammed by tumors show altered RNA and protein content [56]. Platelet counts, platelet–lymphocyte ratios, and mean platelet volume have long been used in cancer diagnosis; RNA-based assays like ThromboSeq are now being explored as novel biomarkers [57,58]. Table 3 summarizes these biomarkers, their isolation and detection methods, and clinical uses.

Subtype-specific variation: Biomarker levels vary across lung cancer subtypes and stages. For example, ctDNA detection is particularly challenging in stage I lung adenocarcinoma (LUAD), with positivity rates as low as 13%, compared to >70% in squamous cell carcinoma (LUSC) [67]. Newer personalized assays that first identify mutations in the tumor and then track them in blood (“tumor-informed” assays) have improved LUAD detection to ~50% even at stage I [68]. In contrast, small cell lung cancer (SCLC) shows high CTC counts, making them especially useful for this subtype [69].

Since no single biomarker captures all tumors, combining multiple biomarkers—such as ctDNA with exosomal RNAs or methylation panels—improves sensitivity and diagnostic accuracy (AUC 0.75–0.82) [70,71]. A practical clinical approach is therefore to rely on ctDNA where shedding is high, and to add exosomal or methylation assays in LUAD, ensuring more balanced detection across all subtypes.

### 4.2. Applications of Liquid Biopsy in Lung Cancer

1. Early Detection: Liquid biopsy enables non-invasive screening, particularly valuable where LDCT is unavailable. The ability to detect tumor-specific mutations, DNA methylation markers, and exosomal miRNAs in easily accessible fluids like blood offers a promising approach to identifying lung cancer at an earlier, more treatable stage [72].
2. Monitoring Response to Treatment: Liquid biopsy allows for frequent sampling, which helps clinicians monitor dynamic changes in ctDNA levels during systemic therapies such as chemotherapy, targeted therapy, and immunotherapy. These changes can guide treatment modifications, predict resistance patterns, and assist in evaluating therapy effectiveness [73,74,75].
3. Prognostic Value: Certain biomarkers offer insight into disease prognosis. Many studies have been done that revealed that patients with eight or more CTCs per 7.5 mL of blood and specific mutations like KRAS G12/G13 are associated with poor prognosis [76,77,78]. Similarly, low levels of tumor-suppressor miRNAs and aberrant DNA methylation patterns also correlate with poor outcomes [79,80].
4. Detection of Metastasis: Liquid biopsy can detect early signs of metastasis by identifying biomarkers released during the metastatic cascade. In cases of leptomeningeal metastasis, cerebrospinal fluid analysis via liquid biopsy detects cancer cells earlier than conventional imaging, aiding in timely intervention [81,82].
5. Monitoring Minimal Residual Disease (MRD): Following curative treatment, liquid biopsy enables longitudinal surveillance through serial ctDNA analysis. Studies show that ctDNA detection within weeks post-treatment can predict molecular relapse well before clinical or radiologic evidence appears, offering a window for early therapeutic action [83,84,85].
6. Tumor Heterogeneity: Liquid biopsy captures spatial and temporal tumor heterogeneity more effectively than a single-site tissue biopsy. It reflects ongoing clonal evolution and acquired resistance mechanisms that emerge under treatment pressure, supporting the personalization of subsequent therapy lines [86,87,88].
7. NSCLC Mutation Profiling: In non-small cell lung cancer (NSCLC), ctDNA facilitates the identification of actionable mutations such as EGFR, ALK, KRAS, and BRAF. This enables the timely initiation or adjustment of targeted therapies, even when tissue biopsy is unavailable or insufficient [89,90].
8. SCLC Mutation Profiling: In SCLC, liquid biopsy has shown unique strengths due to its biology. CTCs are highly abundant and have been used for prognosis, with high baseline counts predicting poor survival [69]. ctDNA analysis can detect hallmark alterations such as TP53 and RB1 loss and track treatment response [91]. Exosomal RNA and protein signatures are also being explored to help distinguish SCLC from NSCLC. While promising, these studies remain limited by small, retrospective cohorts, highlighting the need for larger validation.

### 4.3. Limitations of Liquid Biopsy

Despite its promise, liquid biopsy has important limitations. Sometimes, the biomarker of interest is present in very low concentrations in the peripheral blood, making it difficult to isolate and detect due to technical issues [37]. Another limitation comes in the form of clonal hematopoiesis, which can lead to expansion of mutations in peripheral blood and, therefore, can result in false-positive tests [92]. Finally, a lack of universally standardized protocols for collection, processing, and analysis remains a major barrier [49].

## 5. Role of Artificial Intelligence in Liquid Biopsy

Artificial Intelligence (AI) is playing an increasingly central role in the processing and interpretation of complex biomedical data. In the context of liquid biopsy, AI enables the extraction of meaningful patterns from large, high-dimensional datasets, including circulating tumor DNA (ctDNA), cell-free RNA (cfRNA), and extracellular vesicles (EVs). These analytes provide insights into tumor burden, mutational status, and disease progression, but their complexity often exceeds the capacity of traditional statistical models. By contrast, AI—particularly machine learning (ML) and deep learning (DL)—can identify subtle signals, enhance diagnostic accuracy, and support clinical decision-making [93,94,95,96].

As liquid biopsy technologies generate increasingly large and heterogeneous datasets, the need for sophisticated analytical tools also grows. For example, ctDNA signals may be sparse, while EV cargo or transcriptomic profiles involve thousands of variables. To extract meaningful insights, AI models must handle missing data, variability in feature importance, and nonlinear associations—challenges that are now being addressed through a growing array of algorithmic techniques.

### 5.1. AI Techniques in Liquid Biopsy

Machine learning models such as support vector machines (SVMs), decision trees, and random forest (RF) algorithms have been commonly applied in this field. These models are trained on labeled datasets to recognize patterns and make classifications, such as identifying tumor-derived mutations or distinguishing between cancerous and benign profiles. Among them, RF has become popular due to its ability to handle high-dimensional data and minimize overfitting by averaging the output from multiple decision trees [96,97,98,99,100,101].

Deep learning, a more advanced subset of ML, uses multilayered neural networks to identify intricate relationships within data without needing predefined features. Several DL architectures have proven especially useful in liquid biopsy:

Convolutional Neural Networks (CNNs) are well-suited to recognizing spatial features in structured omics data. Although originally developed for image analysis, CNNs have been adapted for analyzing ctDNA methylation matrices and EV expression profiles. Models like U-Net and 3D CNNs, commonly used in radiology, are now being applied to omics segmentation tasks [102,103,104].

Recurrent Neural Networks (RNNs) and Long Short-Term Memory (LSTM) networks are designed to handle sequential data, making them ideal for tracking biomarker levels over time. These models are especially helpful in evaluating treatment response and predicting recurrence through longitudinal ctDNA monitoring [105,106].

Deep Neural Networks (DNNs) are often used when integrating multiple biological layers—such as genomics, transcriptomics, and proteomics—into one predictive model. This approach is particularly valuable in identifying complex biomarker interactions and disease subtypes [107,108,109,110].

Autoencoders, a type of unsupervised deep learning model, can compress and denoise data, which helps refine signal quality from EV or cfRNA datasets [111].

Generative Adversarial Networks (GANs) are increasingly being employed to synthetically generate new training examples. This is particularly beneficial in rare cancer types where real-world datasets are small or imbalanced. GANs help improve model robustness by expanding the diversity of training data [112,113].

Transfer learning approaches, which repurpose models pre-trained on large datasets (like ResNet or Inception), have shown value in applications where annotated liquid biopsy data are limited. By fine-tuning existing architectures, researchers can achieve strong performance without starting from scratch [114]. All these AI techniques are given in Table 4 along with their uses and applications.

### 5.2. AI in Data Integration and Predictive Modeling

A major advantage of AI lies in its ability to prepare, integrate, and model heterogeneous datasets. Liquid biopsy data are often incomplete, noisy, or variable. AI-driven preprocessing—such as normalization, batch-effect correction, and imputation—improves reliability across cohorts [115]. Once the data are preprocessed, strategies such as cross-validation and grid search are used to tune model parameters and optimize performance.

DL architectures also excel in multi-omics integration. By combining genomic, transcriptomic, and proteomic signals, these models uncover hidden biological patterns. The outputs of such models can be mapped to known biological pathways using tools like Gene Set Enrichment Analysis (GSEA), or aligned with curated resources like KEGG and Gene Ontology (GO) databases, to provide both predictive power and biological interpretability [116,117].

**Table 4:** AI Techniques and Their Applications in Liquid Biopsy.

AI Technique	Application in Liquid Biopsy	Key Strengths	Biomarker Detection Role	Predictive Modeling Role	References
Support Vector Machine (SVM)	Used to classify structured omics datasets and detect disease-linked biomarkers.	Performs well on limited structured data with clear boundaries.	Identifies distinct biomarker profiles from omics-based inputs.	Provides early-stage classification for disease risk.	[96,98]
Random Forest (RF)	Selects and ranks important biological features in large-scale biomarker studies.	Handles noise effectively and reduces overfitting through ensemble learning.	Ranks critical features influencing disease classification outcomes.	Delivers robust prediction of outcomes across diverse datasets.	[99,100,101]
Convolutional Neural Networks (CNNs)	Learns spatial features from ctDNA arrays and EV signal patterns, supporting diagnosis.	Recognizes patterns in structured omics and image-like biological formats.	Highlights spatial characteristics in liquid biopsy data such as EV signatures.	Supports modeling of treatment effects and diagnostic accuracy.	[102,103]
Recurrent Neural Networks (RNNs/LSTM)	Captures time-based changes in biomarker levels for monitoring and prognosis.	Follows sequential biomarker shifts over time, ideal for longitudinal data.	Maps ctDNA fluctuations to biological or clinical changes.	Forecasts recurrence or treatment failure using time-series data.	[105,106]
Deep Neural Networks (DNNs)	Combines multi-omics data into integrated models for improved clinical interpretation.	Builds abstract feature relationships across omics platforms for precise modeling.	Links biomarker patterns across different omics layers.	Stratifies patients and models disease progression based on biomarkers.	[107,108,109]
Autoencoders	Reduces dimensionality and background noise in high-volume datasets without supervision.	Ideal for discovering hidden structures and reducing complexity in noisy datasets.	Uncovers subtle biomarker trends from complex datasets.	Identifies unusual profiles and assists in survival analysis.	[111]
Generative Adversarial Networks (GANs)	Generates synthetic molecular data to improve training where rare biomarker examples exist.	Helps balance datasets through artificial augmentation, especially for rare conditions.	Synthesizes rare variant profiles to enhance model learning.	Improves forecasting when limited real-world data are available.	[112]
Natural Language Processing (NLP)	Extracts meaningful terms from clinical notes and links them to structured biomarker data.	Transforms unstructured clinical text into usable insights that enrich model input.	Connects textual mentions of symptoms or markers to structured data.	Enhances personalized prediction by linking notes and omics.	[98,118]

### 5.3. NLP and Multi-Modal AI Integration

Beyond structured data, AI leverages Natural Language Processing (NLP) to extract features from unstructured clinical text such as electronic health records, pathology reports, and physician notes. NLP can identify symptom clusters, medication histories, or diagnostic cues and integrate them with molecular data, enriching predictive models [98]. When combined with structured omics and imaging inputs, NLP enhances the development of multi-modal models that provide a more comprehensive view of patient status [118]. This enables the development of multi-modal frameworks that combine CNNs for imaging, RNNs for sequential biomarker data, and NLP pipelines for clinical narratives, yielding highly personalized diagnostic systems [106,119,120].

While AI has shown great promise in liquid biopsy, important barriers remain. Chief among them is model transparency: deep learning models often operate as “black boxes.” Techniques such as attention mapping, SHAP values, and LIME provide partial interpretability but do not fully resolve the opacity of neural network decisions. This challenge, discussed further in Section 6.3, underscores the need for explainable AI in clinical adoption.

**Figure 2:**
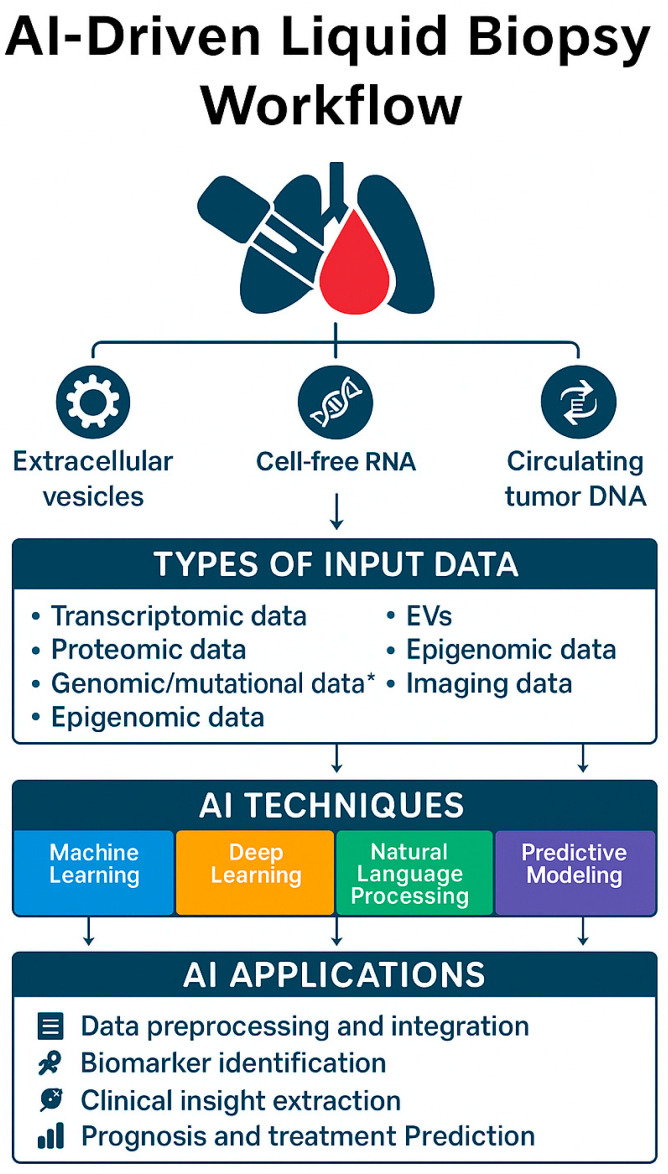
AI-driven liquid biopsy workflow. Biomarkers such as extracellular vesicles, cell-free RNA, and circulating tumor DNA provide multiple data inputs, which are analyzed using AI techniques (machine learning, deep learning, natural language processing, predictive modeling) to support biomarker discovery, prognosis, and treatment prediction. * Genomic/mutational data refer specifically to tumor-derived genetic alterations detectable in cfDNA/ctDNA.

Additionally, real-world implementation will depend on access to high-quality, annotated datasets, as well as regulatory approval for clinical-grade models. These constraints highlight the importance of ongoing standardization efforts and prospective validation. AI Techniques and their applications in liquid biopsy are described in the following Figure 2.

## 6. Use of AI in Liquid Biopsy

Whereas Section 4 focused on the technical methods and integration mechanics of AI, Section 5 shifts to their clinical application. Here we illustrate how AI has been applied in lung cancer care—first in biomarker detection and early diagnosis (Section 6.1), then in monitoring and personalization of treatment (Section 6.2), and finally in multimodal fusion with radiomics (Section 6.3).

### 6.1. For Detection of Specific Biomarkers and Early-Stage Lung Cancer

Over the past decade, NSCLC management has shifted toward genotype-guided care, with EGFR, KRAS, ALK, and TP53 informing targeted therapy eligibility and immunotherapy selection [121,122,123,124]. Although the FDA has approved targeted therapies for at least seven NSCLC genotypes [125], traditional tissue-based methods like FISH and IHC remain invasive, costly, and often inadequate due to tumor heterogeneity and sampling limitations [125]. Liquid biopsy has emerged as a less invasive and more dynamic approach by analyzing circulating tumor DNA (ctDNA) and cell-free DNA (cfDNA) in blood samples. Tests like the cobas^®^ EGFR Mutation Test v2 (Roche Molecular Systems, Pleasanton, CA, USA) have demonstrated high specificity (~98%) in detecting EGFR mutations [126,127], but sensitivity remains a concern in early-stage tumors where ctDNA levels are typically lower [128].

Artificial intelligence (AI) and machine learning (ML) now play a critical role in improving the sensitivity and accuracy of biomarker detection from liquid biopsy data. These technologies can detect subtle genomic and epigenomic patterns that conventional tools may miss and can even integrate imaging (radiomics) and molecular features to non-invasively predict key mutations such as EGFR, ALK, and PD-L1 [129]. Several studies highlight AI’s potential in this domain. Ling et al. applied machine learning to cfDNA methylation patterns and showed that lung cancer could be detected even at very low DNA concentrations [130]. Similarly, a study from China used cfDNA samples with a random forest algorithm to identify EGFR mutation status and support early cancer detection through transcription start site (TSS) analysis [131]. In another application, machine learning models were used to analyze extracellular vesicles (EVs), utilizing a 29-protein plasma-EV signature to distinguish between lung, pancreatic, melanoma, and colorectal cancers [101,132]. Prognostic modeling using support vector machines (SVM) on ctDNA profiles also proved useful in assessing outcomes in post-chemoradiation NSCLC patients [133]. In 2023, an AI system was used to predict PD-L1 expression levels from CT scan images of 385 advanced NSCLC patients, with logistic regression combined with ReliefF emerging as the most accurate model [134]. Additionally, the Orion AI model, trained on circulating non-coding RNAs, achieved 94% sensitivity and 87% specificity for detecting NSCLC through a simple blood test [135].

AI has also significantly enhanced early-stage lung cancer detection, where traditional methods often fall short. For example, Materios et al. used the DELFI model to evaluate cfDNA fragmentation, achieving 94% sensitivity and 80% specificity while reducing false-positive rates by 50% compared to standard low-dose CT [136]. Ray Bahado-Singh et al. integrated DNA methylation data with six machine learning models, further improving diagnostic performance in early disease settings [137]. Likewise, Zhoufeng Wang et al. developed the LunaCAM model using cfDNA methylation profiles to refine risk classification in patients undergoing LDCT, although external validation is still required [138]. In 2024, Luv Purohit et al. applied convolutional neural networks (CNNs) to indeterminate CT nodules, significantly enhancing radiologist performance and achieving 92% sensitivity and 87% specificity [139]. Most recently, Guiyi Ji et al. introduced AI-assisted LDCT-TRAI, which outperformed standard LDCT in identifying early-stage lung cancer, particularly stage I disease, and was associated with improved survival outcomes [140].

Although most AI applications have centered on NSCLC, early studies show potential in SCLC. Chen et al. applied CT-based radiomics to differentiate peripherally located SCLC from NSCLC and achieved good diagnostic accuracy, though their study was retrospective and limited in size [141]. Similarly, Liu et al. developed a CT radiomics model to classify SCLC versus NSCLC, reporting promising performance but emphasizing the need for larger, multi-center validation [142]. Together, these findings suggest that AI can aid in earlier and more accurate recognition of SCLC, but current evidence remains preliminary.

### 6.2. For Monitoring Treatment Response and Personalized Treatment

Beyond initial detection, AI and liquid biopsy also play a crucial role in guiding ongoing therapy. Section 5.2, therefore, explores how these tools enable longitudinal monitoring of response, early relapse detection, and individualized treatment planning.

Accurate monitoring of treatment response is critical for guiding clinical decisions in lung cancer care, and recent advances in liquid biopsy—particularly ctDNA and cfDNA methylation profiling—have significantly improved the precision and timing of disease assessment. Liang et al. demonstrated that patients exhibiting a decline in ctDNA levels during therapy had better outcomes, while elevations in ctDNA often preceded radiographic relapse by weeks or months [130]. Similarly, Hellmann et al. found that the absence of ctDNA after immune checkpoint inhibitor therapy correlated with sustained disease control [143], and Giroux et al. highlighted cfDNA methylation changes as earlier indicators of therapeutic response compared to imaging [144]. Supporting these findings, Widman et al. and Chaudhuri et al. showed that trends in ctDNA closely mirrored disease progression and relapse, with detection occurring up to five months before conventional imaging [145,146]. Helzer et al. further established that genome-wide ctDNA and methylation analysis can track tumor dynamics and emerging resistance across the treatment phases [147].

To enable early detection of minimal residual disease (MRD), high-resolution methylation-based assays such as cfMeDIP-seq and WGBS have been developed, and their integration with machine learning classifiers has allowed for dynamic tracking of relapse risk [28,143,147,148,149,150]. These classifiers interpret cfDNA methylation patterns over time, where declining risk scores typically signal response, while persistently elevated scores often indicate non-response or impending relapse [28,150]. AI-based platforms further elevate this monitoring capability by analyzing ctDNA trends in real-time, offering predictive insights into tumor burden, resistance evolution, and relapse risk [28,144,145,147,149,151] When integrated with imaging data and clinical history, these tools provide a comprehensive, individualized picture of treatment efficacy and the patient’s response [130,143,150].

The same principles extend to AI-driven personalization of treatment and resistance management in lung cancer. ctDNA and cfDNA methylation profiling provide insight into tumor heterogeneity and its evolution under therapy. For example, Chabon et al. used personalized ctDNA assays to detect MRD and predict recurrence in post-surgical NSCLC patients earlier than imaging [149], while Maiti et al. used cfDNA fragmentomics for early-stage tumor detection and proactive planning [150]. Helzer et al. showed that ongoing genomic monitoring could identify resistance mechanisms and support timely therapy adjustments [147]. Assaf et al. developed a multi-analyte blood test combining cfDNA and protein biomarkers to classify lung cancer subtypes and guide more precise treatment selection [28]. Likewise, Abbosh et al. employed phylogenetic ctDNA profiling to track resistant subclones that may benefit from alternative therapies [151].

CfDNA Methylation signatures have also been used to stratify patients by molecular subtype, immune escape characteristics, and likelihood of resistance [146,147,152], allowing physicians to fine-tune therapeutic strategies accordingly [143,148,151]. Persistent methylation abnormalities, even during ongoing therapy, have emerged as early signs of treatment failure and justify escalation or modification of treatment [28,145,149,150]. AI further strengthens this personalization by combining genomic insights from liquid biopsy with clinical data to forecast survival, relapse, and progression risk [130,143,148,150,151]. These platforms not only detect resistance-driving mutations but also identify resistant clones early, enabling timely intervention for rapidly progressing disease [144,147].

The fusion of liquid biopsy, advanced epigenetic profiling, and AI-powered analytics marks a paradigm shift in precision oncology, enabling earlier detection, more dynamic monitoring, and truly individualized treatment in lung cancer care and are summarized in Table 5.

### 6.3. Integration of AI, Liquid Biopsy, and Radiomics for Multimodal Early Lung Cancer Detection

While Section 5.1 and Section 5.2 focused on liquid biopsy in isolation, emerging work increasingly integrates biopsy data with imaging features. Section 5.3 highlights these multimodal fusion approaches, showing how AI can combine molecular and radiomic signals to further refine early detection and monitoring.

Liquid biopsy contributes molecular specificity (ctDNA mutations, cfDNA methylation/fragmentomics, EV and ncRNA signatures), whereas radiomics captures spatial phenotype and heterogeneity from CT/LDCT. Separately, each modality has gaps—ctDNA sensitivity is limited in small tumors; radiomics can inflate false positives and is protocol-dependent. AI provides the fusion layer that jointly models complementary signals to stabilize performance.

Evidence of benefit- The LunaCAM model fused cfDNA methylation profiles with LDCT imaging, improving specificity for indeterminate pulmonary nodules and reducing false positives compared to imaging alone [138]. Assaf et al. demonstrated that multi-analyte liquid biopsy combining cfDNA and protein biomarkers with machine learning enhanced diagnostic accuracy beyond single-biomarker assays [151], underscoring the value of molecular integration that could be extended to imaging. More recently, Zhang et al. combined CT features with EV long RNA signatures to distinguish early-stage lung adenocarcinoma, showing superior performance compared to either modality alone [153]. In therapeutic contexts, ctDNA integrated with radiomics features improved the prediction of treatment response and recurrence in NSCLC patients undergoing chemoradiation. Smaller exploratory studies also suggest added value: Lafata et al. reported that CT radiomic heterogeneity combined with cfDNA profiles correlated more strongly with survival and treatment response in locally advanced NSCLC [154], while Yousefi et al. showed that integrating radiomic phenotypes with cfDNA mutation data and clinical variables improved prognostic model performance (c-statistic ≈ 0.83) [155]. Beyond lung cancer, radiogenomic studies linking imaging phenotypes to genomic and transcriptomic profiles provide additional precedent for cross-domain fusion strategies [156].

**Figure 3:**
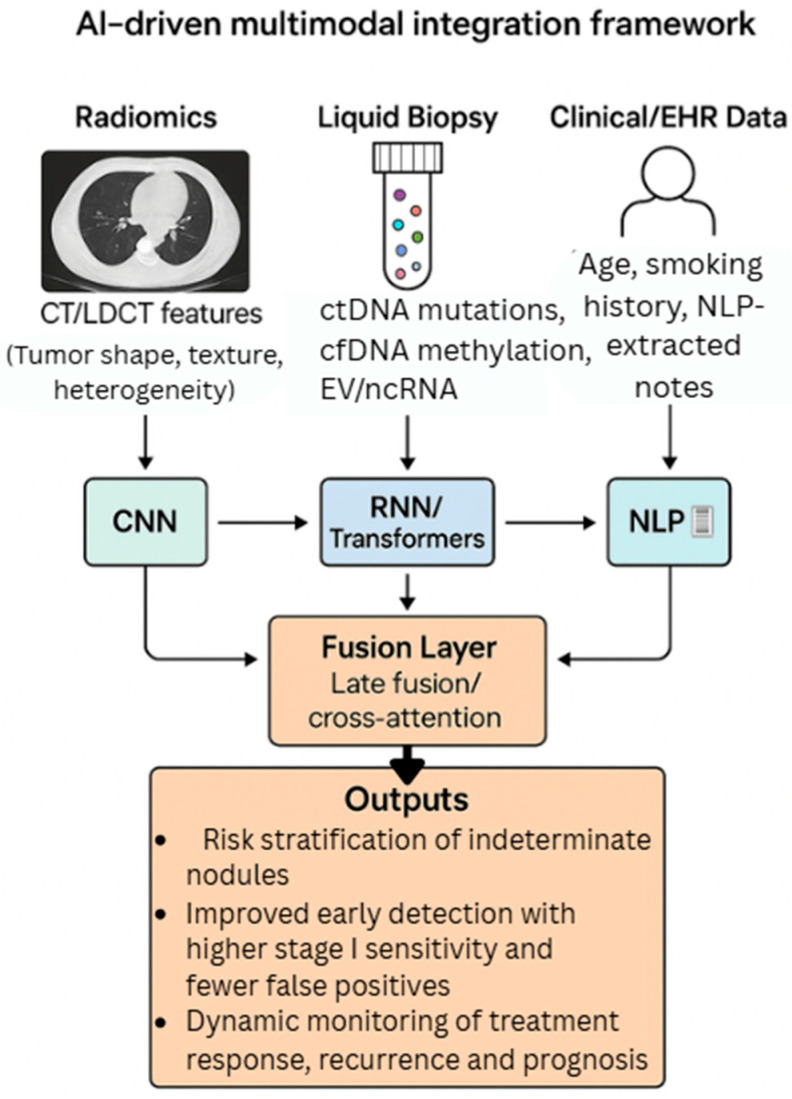
AI-driven multimodal integration framework for lung cancer detection and monitoring. Radiomics (CT/LDCT features such as tumor shape, texture, and heterogeneity), liquid biopsy (ctDNA mutations, cfDNA methylation, extracellular vesicles, and ncRNA signatures), and clinical/EHR data (age, smoking history, NLP-extracted notes) are processed in parallel through a multimodal deep learning pipeline. Separate encoders (CNN for imaging, RNN/transformers for biomarkers, NLP models for clinical narratives) generate feature representations, which are fused at the decision layer to produce calibrated predictions. Integrated outputs enable risk stratification of indeterminate nodules, improved early detection with higher Stage I sensitivity and fewer false positives, and dynamic monitoring of treatment response, recurrence, and prognosis.

Building on prior radiogenomic and biomarker-fusion studies, we outline a conceptual framework tailored for lung cancer that integrates radiomics, liquid biopsy, and AI within a unified clinical workflow. This framework is intended as a synthesis to guide future multimodal research and translational implementation. This integrative workflow is illustrated in Figure 3, which depicts how radiomics, liquid biopsy, and clinical/EHR data can be processed in parallel and fused within an AI multimodal model to yield clinically actionable outputs.

Proposed framework –

We propose a multi-branch deep-learning pipeline in which (i) radiomics (shape/texture/heterogeneity; optional deep image embeddings) and (ii) liquid-biopsy features (mutations, methylation/fragmentomics, EV/ncRNA signatures) are encoded in parallel, optionally alongside (iii) structured clinical variables and NLP-extracted narrative features (smoking history, prior nodules). Learned representations are fused at a decision layer (late fusion) or via cross-attention/transformers (mid-fusion) to yield calibrated risks for: (a) malignancy of indeterminate nodules, (b) early detection in screening cohorts, and (c) short-interval recall versus routine follow-up. In screening, an operational workflow could be: LDCT → AI-radiomics pre-risk → reflex targeted liquid biopsy for the gray-zone nodules → multimodal AI final risk → management (surveillance, diagnostic CT/PET, or biopsy).

Maturity and gaps: Most integration studies are small, retrospective, and heterogeneous, with limited external validation. Nonetheless, AI-mediated fusion consistently reduces false positives and boosts sensitivity for small-volume disease, offering a more holistic picture than any single modality. Prospective, multi-center trials with standardized pipelines are the next step to establish clinical value. As methods mature, such frameworks are expected to redefine lung cancer risk stratification and early detection strategies.

All the studies summarized in Section 5 recurring constraints include retrospective single-center designs, small or homogeneous cohorts, and diverse technical pipelines that hinder reproducibility and cross-study comparison. Few head-to-head benchmarks vs. LDCT or tissue biopsy exist, limiting claims of incremental value. Prototype frameworks (e.g., LunaCAM, multi-analyte assays, multimodal fusion) are promising but await large-scale external validation. These evidence gaps motivate the systemic solutions discussed in Section 6 (standardization, interpretability, equitable access, and comparative effectiveness).

## 7. Challenges and Limitations of AI in Liquid Biopsy

### 7.1. Data Quality and Standardization

The quality of samples used for liquid biopsy can vary widely depending on how they are collected, stored, and handled. Hospitals and labs often use different types of collection tubes containing various preservatives. If the wrong tube or preservative is used, cells can undergo lysis, contaminating the sample. The time between collection and processing also plays a critical role—delays can lead to degradation of DNA or RNA. Similarly, improper temperature during storage or transport may damage the sample. Even the centrifugation step—its speed, time, and temperature—must be precisely controlled to avoid contamination. Different labs may use varying DNA/RNA extraction methods, which can affect what’s actually detected. If these procedures differ across hospitals, the data quality becomes inconsistent, making it difficult for AI models trained on one dataset to perform accurately in another setting. This lack of standardization remains a major barrier to reliable AI applications in liquid biopsy [157].

Multi-center initiatives have attempted to address this barrier. For example, the BloodPAC Consortium developed shared standards for liquid biopsy sample collection and analysis to enable reproducibility across labs [158]. Similarly, the TRACERx study standardized tissue and liquid biopsy protocols across multiple UK hospitals for consistent data integration [159]. In imaging-based biomarkers, radiomics harmonization efforts such as ComBat have been applied to reduce inter-scanner variability in multi-site studies [160]. While further refinement is needed, these preliminary successes highlight the feasibility of reducing variability through unified methodologies. Linking these ongoing efforts to broader calls for standardized pipelines (as discussed in Section 7) strengthens the case for scalable clinical translation.

### 7.2. Ethical and Privacy Concerns

Applying AI in healthcare, especially in liquid biopsy for lung cancer, raises several ethical and privacy questions. Key concerns include: Who owns the patient-derived data? How is the data being used? Has the patient given informed consent for these uses? Any use of patient data in AI models must comply strictly with privacy laws like HIPAA and GDPR. Electronic Health Records (EHRs), which are often used for training AI, contain sensitive personal information, including diagnoses, medications, and test results. There’s growing concern about how this data might be accessed or used by third-party companies that may not prioritize patient welfare. Another issue is how AI-generated predictions or decisions are applied in real clinical settings, and whether patients have a say in that process [161].

### 7.3. Model Transparency and Interpretability

As noted earlier (line 453), model transparency refers to the clarity with which clinicians can understand an AI system’s reasoning process. Many AI models, especially deep learning systems, are often considered “black boxes” because they produce outputs without clear explanations. In medicine, this lack of interpretability is a major concern—clinicians need to understand how a decision or prediction was made. This is where Explainable AI (XAI) becomes essential. Methods such as SHAP (Shapley Additive Explanations) [162] and LIME (Local Interpretable Model-agnostic Explanations) [163] provide ways to assign feature importance or approximate local decision boundaries. However, these tools only deliver partial insights and do not resolve the fundamental black-box nature of deep neural networks. Their explanations are often statistical rather than mechanistic, which limits clinical utility. As pointed out in a critical review, many explainability methods provide “false reassurance” without offering actionable understanding for clinicians [164]. Thus, while XAI represents progress toward transparency, its current role in clinical liquid biopsy remains supplementary, not definitive. Since clinical decisions depend heavily on data context, doctors must remain the final filter to evaluate whether an AI-based recommendation is valid and safe to act on [165]. A recent meta-analysis on explainable AI in clinical decision support further underscores this challenge, highlighting both the promise and the usability gaps of current XAI methods in healthcare and emphasizing that improved interpretability is essential for trustworthy clinical adoption [166].

### 7.4. Cost and Accessibility

Bringing AI-powered liquid biopsy tools into routine clinical care is challenging due to high costs and limited compatibility with existing infrastructure. While liquid biopsy holds great potential for early detection and monitoring of cancer by analyzing ctDNA, widespread adoption remains difficult in resource-constrained settings. Some studies suggest liquid biopsy may lower overall costs by reducing the need for invasive tissue biopsies [167], but the initial investment is significant. This includes the cost of advanced sequencing platforms, data processing systems, and specialized training for personnel. Techniques like next-generation sequencing (NGS) and advanced ctDNA analysis increase both cost and technical complexity [168,169].

These challenges are particularly acute in low- and middle-income regions, where shortages of LDCT equipment, limited laboratory infrastructure, and fewer trained specialists constrain the feasibility of advanced AI–liquid biopsy pipelines. Pragmatic strategies such as dried blood spot collection, pooled batching of samples, and regional centralization of assays have been discussed as feasible adaptations in resource-limited settings. However, most of these strategies remain at the pilot or conceptual stage, and their scalability, cost-effectiveness, and diagnostic reliability still require systematic evaluation through larger multi-center studies.

### 7.5. Population Generalizability and Comparative Effectiveness

Most AI–liquid biopsy studies have been conducted in relatively homogeneous populations recruited from large academic medical centers. Such cohorts often lack ethnic diversity, broad comorbidity profiles, and representation from varied healthcare settings, which limits the generalizability of findings to real-world clinical practice. In addition, while individual studies demonstrate promising diagnostic performance, to date no studies have directly compared AI-enhanced liquid biopsy with standard modalities such as LDCT or tissue biopsy within the same patient cohorts. This absence of head-to-head evidence prevents firm conclusions about comparative accuracy, cost-effectiveness, and patient impact, underscoring the need for broader validation in multi-ethnic, multi-center, and community-based populations.

## 8. Future Directions and Research Opportunities

For AI to be successfully implemented in clinical settings, one key prerequisite is the availability of large, well-annotated datasets. Integration of multi-omics data—genomic, transcriptomic, proteomic—with imaging and liquid biopsy data could improve prediction accuracy, sensitivity, and specificity, while reducing false positives [170]. However, much of the publicly available biomedical data remains insufficiently annotated, hindering its utility in machine learning model development [171]. Another critical methodological challenge is class imbalance, particularly pronounced in early lung cancer datasets where positive cases are scarce. To mitigate this, advanced oversampling and augmentation methods such as SMOTE have been applied, with reports showing improvements in classification accuracy from ~74% to ~94% in cancer classification tasks [172]. Similarly, hybrid approaches like K-Means SMOTE combined with neural networks have demonstrated >93% accuracy and AUC values >96% in lung cancer risk prediction [173]. While these results are promising, they remain limited by retrospective, single-center datasets and risk introducing synthetic artifacts that may not reflect true biological variability. Future research should focus on validating such augmentation techniques in larger, multi-center, and prospectively collected datasets. Integrating synthetic resampling with domain-informed biological priors, or coupling it with federated learning frameworks, may provide more clinically reliable solutions for overcoming data imbalance in liquid biopsy AI models.

In the era of precision medicine, AI is increasingly applied for modeling drug–protein interactions, identifying drug resistance mechanisms, and constructing 3D protein structures [174]. Additionally, liquid biopsy has emerged as a minimally invasive alternative to tissue biopsy for tracking tumor evolution and heterogeneity over time. This method enables serial sampling of ctDNA in patients with advanced metastatic cancer, allowing for better real-time monitoring of tumor mutational dynamics [175]. AI tools are especially suited for handling such complex, high-volume datasets, uncovering patterns that traditional statistical methods may miss. Another emerging opportunity lies in the synergy between radiomics and AI. Radiomics involves extracting quantifiable features from imaging data such as tumor texture and heterogeneity. AI models can automate this process and predict clinical outcomes from large-scale imaging datasets without manual input [176]. Combining radiomics with liquid biopsy could improve tumor characterization and treatment stratification. Moreover, radiogenomic AI models are being explored to overcome tumor heterogeneity—an ongoing barrier to personalized therapy. For instance, CT radiomics has been shown to distinguish between primary lung tumors and metastases with an AUROC of 0.92 [177], while liquid biopsy allows for estimating tumor mutational burden (TMB) through next-generation sequencing and machine learning analysis of ctDNA, cfDNA, and circulating tumor cells [2]. These tools could significantly enhance therapeutic targeting and patient-specific treatment planning. To make these technologies truly effective, future research should focus on collaboration between institutions and standardizing data methods, so that AI tools can be used reliably, on a larger scale, and in real-world clinical practice. To translate AI–liquid biopsy tools into routine clinical care, future research must prioritize validation in diverse, multi-ethnic, and multi-center populations that reflect real-world heterogeneity in comorbidities and healthcare settings. Equally important are comparative effectiveness studies that evaluate AI–liquid biopsy not in isolation, but alongside standard modalities such as LDCT and tissue biopsy. Such trials should incorporate clinically meaningful endpoints including diagnostic accuracy, cost-effectiveness, and patient-centered outcomes such as tolerability and psychological impact. Integration frameworks that combine AI-driven liquid biopsy with LDCT screening represent a particularly promising avenue, but require rigorous prospective benchmarking to establish incremental value. Looking ahead, a critical step will be the establishment of standardized pipelines for liquid biopsy and AI analysis across institutions. Harmonized procedures for sample collection, processing, and data annotation would not only reduce variability but also enable the creation of robust, multi-center datasets for AI model development. Embedding these pipelines into international collaborations will be key to moving from promising single-center studies toward scalable, real-world clinical translation. Future research should also focus on adapting AI–liquid biopsy workflows for low-resource regions, where simplified approaches such as dried blood spot collection and centralized analysis could expand access to early detection.

Taken together, these opportunities highlight both the promise and the current limitations of AI-driven multimodal integration. While new innovations and validation studies continue to advance, the field now requires a clear roadmap for translation into practice.

### Concluding Remarks

While technical progress has been impressive, the integration of AI, liquid biopsy, and radiomics remains at an early stage. A few priorities stand out for translating these tools into practice: (i) building large, diverse datasets through federated collaboration, (ii) advancing multimodal fusion with methods such as transformers and graph neural networks, (iii) conducting head-to-head trials that benchmark hybrid workflows against current standards such as LDCT, PET/CT, and tissue biopsy, (iv) ensuring interpretability so clinicians can trust model outputs, and (v) evaluating cost and accessibility in real-world health systems. Taken together, these steps form a practical roadmap for moving from proof-of-concept models to reliable, clinically adopted strategies for early detection and personalized lung cancer care.

## 9. Conclusions

Artificial Intelligence, when integrated with liquid biopsy and radiomics, holds great promise for transforming lung cancer care. Together, these technologies can support earlier diagnosis, better monitoring of disease progression, and more precise treatment selection. Their non-invasive nature, combined with the ability to process large volumes of complex data, provides an opportunity to improve outcomes while reducing patient burden. However, to fully realize these benefits, certain challenges must be addressed. These include the need for high-quality, standardized data, greater model transparency, and strong ethical safeguards. With continued collaboration among researchers, clinicians, and technology developers, AI-driven tools can become a practical and reliable part of routine cancer care, making personalized medicine more accessible and effective for lung cancer patients in the years ahead.

## Figures and Tables

**Table 1:** Subtypes of SCLC with dominant transcription factors and neuroendocrine features.

Subtype	Dominant Transcription Factor	Neuroendocrine Features
SCLC-A	ASCL1	High [25]
SCLC-N	NEUROD1	Intermediate [25]
SCLC-P	POU2F3	Non-neuroendocrine [25]
SCLC-Y	YAP1	Associated with Chemoresistance [25]

**Table 2:** Common actionable mutations and molecular targets in non-small cell lung cancer (NSCLC), with associated pathways and their diagnostic, prognostic, or therapeutic roles.

Mutation/Target	Pathway	Clinical Implication
KRAS (G12C/D/V)	RAS–RAF–MEK–ERK	Risk factor associated with tobacco exposure [27]
BRAF V600E	MAPK pathway	Predictive biomarker; targetable with BRAF/MEK inhibitors [27]
HER3	PI3K signaling via dimerization	Predictive biomarker; linked to resistance to EGFR inhibitors [17]
Nectin-4, ITGB6	Adhesion, immune suppression	Prognostic biomarker; promotes migration and immune evasion [17]
FRα	JAK/STAT3 and ERK	Predictive biomarker; enhances proliferation [17]
PRMT5	Epigenetic silencing	Therapeutic target; especially active in MTAP-deleted tumors [28]

**Table 3:** Key biomarkers in lung cancer detected via liquid biopsy.

S. No.	Biomarkers	Isolation Methods	Detection Methods	Uses
1.	Circulating tumor cells (CTCs)	CellSearch system (EpCAM-based) [59,60]	Immunocytochemistry, FISH [60]	metastasis
2.	Circulating tumor DNA (ctDNA)	DNA extraction (Qiagen Circulating Nucleic Acid Kit) [61]	Droplet digital, PCR, NGS [47,62]	detecting mutations, prognostic
3.	Extracellular vesicles (EV)	Precipitation (miRCURY), Size-exclusion chromatography (qEV, Exo-spin) [63]	Quantitative PCR, NGS [63]	metastasis
4.	MicroRNAs (miRNAs)	Phenol-chloroform extraction (TRIzol method) [64]	Quantitative reverse transcription PCR	prognostic
5.	DNA methylation markers	Bisulphite conversion, The Infinium HumanMethylation450 BeadChip array, The HumanMethylationEPIC BeadChip [55,65].	Methylation-specific PCR	screening, diagnostic
6.	Tumor-educated platelets (TEPs)	Slow and fast centrifugation	ThromboSeq [66]	Emerging biomarker

**Table 5:** Summary of key studies utilizing artificial intelligence (AI) in combination with liquid biopsy techniques for lung cancer detection, molecular profiling, treatment monitoring, and response prediction. The table highlights study years, AI approaches, liquid biopsy biomarkers, clinical applications, outcomes, and notable limitations.

Year & Author	Biomarker/Target	Method/AI Approach	Validation Cohort Characteristics	Clinical Application	Results & Limitations
2024, Bie et al. [131]	cfDNA methylation	cfDNA + ML (XGBoost, SVM)	196 pts (96 LC, 100 HC); external 142 samples; train/valid split	Early NSCLC detection	High accuracy incl. early stage; benign specificity underexplored; multi-center validation needed
2020, Hoshino et al. [101]	EV proteins	Plasma EV + ML signature	497 EVP samples; 426 human; 152 control, 274 cancer across fluids	Pan-cancer incl. lung	13-protein EV panel; high sensitivity/specificity; lung-specific metrics lacking
2023, Asleh et al. [132]	PD-L1 in NSCLC	CT radiomics + Logistic Regression	Retrospective imaging dataset; no ext. validation	PD-L1 prediction	Accurate vs. radiologists; retrospective only
2023, Wu et al. [133]	ncRNAs in NSCLC	Orion AI (deep learning)	59 NSCLC pts, 97 plasma samples, 3 timepoints, China	Early detection & subtyping	Sens 94%, Spec 87%; prospective validation needed
2023, Yolchuyeva et al. [134]	EGFR, KRAS, ALK, BRAF	cfDNA + AI classifier	385 NSCLC pts; stratified by gender, age, smoking, ECOG, PD-L1, PFS	Mutation detection	High concordance with tissue; rare mutations less reliable
2024, Karimzadeh et al. [135]	cfDNA fragments + mutations	Fragmentomics + UMI-NGS + ML ensemble	1050 (419 NSCLC, 631 control); 80% training	Early detection & subtyping	High specificity; reduced false positives; requires deep seq/infra
2024, Purohit et al. [139]	Indeterminate nodules	LDCT CNN reanalysis	Retrospective LDCT dataset; no external validation	Early lung CA diagnosis	Sens 92%, Spec 87%; ext. validation pending
2021, Mathios et al. [136]	cfDNA methylation	Bisulfite seq + ML	365 at-risk indiv.; external validation 385 controls + 46 LC pts	Early NSCLC detection	High sensitivity; distinguishes benign vs. cancer
2022, Bahado-Singh et al. [137]	cfDNA methylation	Multi-ML ensemble	10 cases vs. 20 controls, all Caucasian; 10-fold cross-validation	Risk prediction	Improved early detection; retrospective; small
2023, Wang et al. [138]	cfDNA methylation (LunaCAM)	ML classifier + LDCT	Discovery 429, training 513, validation 172	Risk stratification	Improved specificity; real-world validation needed
2025, Ji et al. [140]	Early-stage lung CA	AI-augmented LDCT-TRAI	259,121 screened; 87,260 positive; 728 LC dx (634 non-smokers)	Detection & outcomes	Enhanced Stage I detection, survival benefit; multi-site perf. unclear
2021, Liang et al. [130]	ctDNA in lung cancer	NGS profiling	Training/validation details not reported	Tumor burden & relapse	ctDNA decline = response; early rise = progression; subtype coverage lacking
2020, Hellmann et al. [146]	ctDNA in NSCLC	NGS + ML risk model	31 advanced NSCLC pts; no external validation	ICI response prediction	ctDNA clearance = durable benefit; retrospective only
2020, Giroux et al. [143]	cfDNA methylation	Targeted profiling + ML	79 NSCLC pts; no separate validation set	Immunotherapy monitoring	Profiles distinguished responders early; small sample
2020, Jee et al. [144]	cfDNA methylation	cfMeDIP-seq + ML	1127 NSCLC pts; no external validation subset	MRD & relapse detection	Sens ~85%, Spec ~95%; lung-specific validation absent
2019, Chaudhuri et al. [145]	ctDNA (lung & colorectal)	Deep targeted seq + ML	40 lung CA pts + 54 HC; internal validation only	Response & relapse	93% concordance w/imaging; relapse detected ~5 mo earlier
2020, Chabon et al. [147]	ctDNA (resected NSCLC)	Tumor-informed NGS	Validation details not reported	MRD & relapse	Relapse detected ~5 mo pre-imaging; requires tumor tissue
2024, Sujit et al. [148]	cfDNA fragmentation	Fragmentomics + ML	394 pts: discovery 199, validation 195; external cohort	Early NSCLC detection	Acc 93% (AUC = 0.93); incl. small tumors; specificity varies
2021, Widman et al. [149]	ctDNA in NSCLC	Serial NGS ctDNA	Validation details not reported	ICI monitoring	ctDNA decline = better survival; early rise = relapse
2021, Helzer et al. [150]	ctDNA + CNVs	Whole-genome cfDNA + ML	UW (*n* = 320), GRAIL (*n* = 198); train/valid split	Broad monitoring	Captured burden & resistance pre-imaging; costly/complex
2023, Assaf et al. [151]	cfDNA + proteins	Multi-analyte ML blood test	1954 samples; 466 NSCLC pts; ext. validation in 73 OAK trial pts	Early lung CA detection	Sens 89%, Spec 99%; better than single markers; validation pending
2017, Abbosh et al. [152]	ctDNA mutations	Phylogenetic tracking	Validation details not reported	Post-op relapse detection	Relapse ID up to 11 mo earlier; resource-intensive

## Data Availability

This review was based on publicly available academic literature databases.
